# Relationships Between Parent Ratings of Attention-Deficit/Hyperactivity Disorder Behaviors and the Virtual Reality Attention Tracker in School-Aged Children: Cross-Sectional Study

**DOI:** 10.2196/76673

**Published:** 2025-10-24

**Authors:** Yuyin Bai, Yange Luo, Crystal C W Goh, Cuiziyi Rui, Rui Gao, Yingying Wu, Zhongmei Jiang, Lifeng Lu, Albert “Skip” Rizzo, Bo Bi

**Affiliations:** 1 Department of Clinical Psychology, The Eighth Affiliated Hospital of Sun Yat-sen University, 3025 Shenzhen Middle Road, Shenzhen, 518033, China, 86 83982222-60375; 2 Department of Ophthalmology, Yong Loo Lin School of Medicine, National University of Singapore, Singapore, Singapore; 3 Neurobiology Program, Life Science Institute, National University of Singapore, Singapore, Singapore; 4 Cognitive Leap Solutions Inc, Irvine, CA, United States; 5 Duke Kunshan University, Kunshan, Jiangsu, China; 6 Shanghai Cognitive Leap Center Limited, Shanghai, China; 7 California Institute of Health, Irvine, CA, United States; 8 Wiseheart Technology Company, Shanghai, China; 9 University of Southern California Institute for Creative Technologies, Los Angeles, CA, United States

**Keywords:** attention-deficit/hyperactivity disorder, Virtual Reality Attention Tracker, continuous performance tests, parent ratings, ADHD, VRAT, CPT

## Abstract

**Background:**

Attention-deficit/hyperactivity disorder (ADHD) is a common neurodevelopmental disorder with symptoms of inattention, hyperactivity, and impulsivity, which can significantly impact social functioning. Traditional ADHD diagnostic methods rely on subjective behavioral ratings and neuropsychological tests, but these tools may have limitations, including biases and restricted ecological validity.

**Objective:**

This study aimed to explore the relationship between parental ratings of ADHD symptoms and performance on a Virtual Reality Attention Task (VRAT) in school-aged children. The objective was to examine whether the VRAT could provide an objective, ecologically valid measure of ADHD symptoms, and how it correlates with established ADHD rating scales, namely the Swanson Nolan and Pelham, Version IV Scale-Parent Scales (SNAP-IV) and the Chinese version of the Conners’ Parent Rating Scale–Revised (CPRS-48).

**Methods:**

A total of 425 school-aged children (6 to 8 years old) participated in this cross-sectional study. They completed the VRAT, a continuous performance test embedded within an immersive virtual classroom, while their parents completed the SNAP-IV and CPRS-48 rating scales. Bivariate correlational analysis and receiver operating characteristic curve analysis were used to examine the relationships between the VRAT and rating scales, as well as the discriminatory capacity of the VRAT.

**Results:**

VRAT shows several correlations with the SNAP-IV subscales and the CPRS-48 subscales. Participants with higher SNAP-IV inattention and hyperactivity scores exhibited lower VRAT attention and motion performance index scores (all *P*<.001). For the CPRS-48, significant correlations were noted with conduct problems, learning problems, psychosomatic problems, impulsivity-hyperactivity, and attention index, particularly with the attention performance and motion performance indexes (all *P*<.05). Gender differences were significant in attention and hyperactivity measures of the SNAP-IV and CPRS-48, while only latency showed gender differences in the VRAT. In addition, the receiver operating characteristic analysis indicated that the key performance metrics of VRAT showed moderate discriminatory power, with area under the curve values varying from 0.56 to 0.74.

**Conclusions:**

This study highlights the potential of virtual reality–based assessments such as the VRAT in ADHD diagnostics, providing an innovative approach to evaluating attention in a more immersive and ecologically valid setting. However, given the modest correlation with parent rating scales, it suggests that a combination of objective and subjective assessment tools would provide the most accurate and comprehensive ADHD diagnosis.

## Introduction

Attention-deficit/hyperactivity disorder (ADHD) is a neurodevelopmental disorder characterized by persistent inattention, impulsivity, and hyperactivity, significantly impacting daily functioning [1,2]. Currently, the diagnosis of ADHD relies on comprehensive clinical evaluation conducted by qualified professionals, who integrate subjective behavioral reports from parents and teachers with objective neuropsychological test results, such as continuous performance tests (CPTs) [3]. Widely used rating scales—including the Swanson Nolan and Pelham, Version IV Scale-Parent Scales (SNAP-IV) and the Chinese version of the Conners’ Parent Rating Scale–Revised (CPRS-48)—serve as key tools for determining whether individuals meet ADHD diagnostic criteria [4,5]. These scales also demonstrate utility in identifying comorbidities (eg, substance use disorders, anxiety, and antisocial behavior) and predicting disorder persistence longitudinally, as they correlate strongly with other diagnostic measures [3,6]. Despite their widespread clinical application, rating scales are not without limitations. Assessments based on parent and teacher reports are susceptible to rater bias, and although many of these scales demonstrate strong psychometric properties, their predictive validity for treatment outcomes remains limited [7].

Among objective tools, CPTs have become a cornerstone of ADHD assessment, measuring sustained attention, impulsivity, and response inhibition using computerized target and nontarget paradigms [8,9]. Performance metrics derived from CPTs—such as omission and commission errors—are useful in distinguishing individuals with ADHD from neurotypical peers and in monitoring treatment response [10]. However, traditional CPTs have been criticized for insufficient sensitivity in detecting distractibility, a core symptom in ADHD. Most commonly used CPTs are administered in controlled environments that minimize external distractions—conditions that do not reflect the real-world attentional challenges faced by children with ADHD [11]. Furthermore, the ecological validity of neuropsychological tests is often questioned, particularly when borderline or mildly impaired scores fail to predict real-life functional outcomes [1,12]. In addition, CPTs have a reputation for eliciting negative reactions in youngsters and are frequently seen as monotonous. Consequently, numerous scholars have advocated for enhancements to address the deficiency of ecological validity in CPTs, which is crucial for predicting real-life functioning in assessments of attentional processes [11].

The Virtual Reality Attention Tracker (VRAT), an AX-type CPT embedded within a virtual classroom environment, has emerged as a promising alternative for ADHD assessment. VRAT integrates immersive, ecologically valid stimuli and motion-tracking technology to objectively evaluate attention, impulsivity, and hyperactivity [13,14]. Findings have demonstrated that virtual reality (VR)–based assessments hold promise for the neuropsychological evaluation of cognitive processes such as executive functions, memory, visuospatial analysis, and everyday functioning [1]. Importantly, these VR-enabled measures have been shown to discriminate effectively between cognitively healthy individuals and those with impairments and could elevate the motivation of children to participate more actively [15,16].

This study aims to assess the validity of VRAT by examining its correlation with traditional ADHD assessment tools and evaluating its ability to distinguish children with ADHD from typically developing peers. The study investigates potential gender differences in ADHD assessment outcomes across VRAT and traditional tools. Finally, we compare the sensitivity and specificity of VRAT in identifying at-risk individuals, exploring its potential to enhance engagement and diagnostic accuracy. By addressing these objectives, our findings are expected to contribute to a more precise and ecologically valid assessment of ADHD, ultimately improving clinical diagnosis and personalized intervention strategies.

## Methods

### Study Design and Participants

This cross-sectional study evaluated the comparative validity of the VRAT against established ADHD assessment tools (SNAP-IV and CPRS-48) in school-aged children. Participants were recruited from Shenzhen Hongling Primary School, a public school located in an urban area of Shenzhen, a major first-tier city in China, from November 11 to December 10, 2022. Most students come from ordinary urban families, so the sample reflects the population typically served by public schools in large cities. A total of 507 children aged 6‐8 years were initially enrolled, with 425 completing all assessments after exclusions. Three licensed psychologists supervised the assessment battery, ensuring accurate administration and data collection.

Demographic information, including age, gender, and parental education level, was collected via a standardized questionnaire completed by parents alongside the SNAP-IV and CPRS-48 scales. The questionnaire captured child age (in years), gender (male or female), and parental education (categorized as primary, secondary, or tertiary education). Inclusion criteria were (1) enrollment in the participating elementary school, (2) no known neurological disorders or visual impairments affecting VRAT performance, and (3) informed consent from parents and school principals. Exclusion criteria included (1) requiring more than 3 VRAT practice sessions, (2) incomplete VRAT performance or inability to understand the task, (3) CPRS-48 questionnaires with completion times greater than 30 minutes, which also had a standardized completion time (Z-score) less than –1, and (4) long-string analysis responses with a Z-score greater than 1.5. Participation was voluntary, with no compensation offered, and anonymity was ensured by removing identifiable information from collected data. Textbox 1 lists inclusion and exclusion criteria for a cross-sectional study of ADHD assessment in school-aged children.

Textbox 1. **Inclusion and exclusion criteria for a cross-sectional study of attention-deficit/hyperactivity disorder assessment in school-aged children.**

**Inclusion criteria**

- Enrolled in Shenzhen Hongling Primary School
- Aged 6‐8 years
- No known neurological disorders or visual impairments affecting Virtual Reality Attention Tracker (VRAT) performance
- Provided informed consent from parents and school principals

**Exclusion criteria**

- Required more than 3 practice sessions to understand the VRAT task
- Did not complete the VRAT or was unable to understand the task
- CPRS-48 (the Chinese version of the Conners’ Parent Rating Scale–Revised) questionnaires taking >30 minutes (outliers, Z-score <1 SD from mean)
- Long-string analysis: continuous choices >1.5 SD from the mean

Sample size was calculated based on detecting moderate correlations (*r*=0.30) between VRAT and parent rating scales with 80% power and a 0.05 significance level, requiring a minimum of 400 participants. We recruited 507 participants to account for potential exclusions, yielding a final sample of 425, which was sufficient for the planned statistical analyses. While convenience sampling may introduce selection bias, the sample size ensured adequate statistical power.

For this study, participation was voluntary, and informed consent was obtained from both parents and school principals after they were provided with detailed information about the study’s objectives, procedures, potential risks and benefits, and the option to decline participation. While convenience sampling offers practicality and efficiency, it may introduce selection bias and limit the generalizability of our findings. Despite these limitations, this approach was suitable for our study’s objectives and allowed us to gather a sufficient sample size of 425 participants, which was adequate for our intended statistical analyses.

### Study Outcomes

The primary outcome of this study is the correlation coefficient between the VRAT scores and the scores from the SNAP-IV and CPRS-48 assessments, reflecting the degree of association between VRAT performance and ADHD symptom severity as gauged by SNAP-IV and CPRS-48. Participants underwent the VRAT, a computerized attention task, and parents completed the SNAP-IV (Chinese version) and CPRS-48 questionnaires. Demographic factors such as gender are considered potential predictors, while comorbid conditions and parental education level are regarded as possible confounders that may influence the correlation between VRAT results and questionnaire scores.

### Data Measures

The study used the SNAP-IV and CPRS-48 as representative ADHD assessment tools. The SNAP-IV is a 26-item questionnaire designed to evaluate the symptoms and severity of ADHD [17]. It is completed by parents. The questionnaire includes 18 items focused on ADHD symptoms (items 1 through 9 address inattention, while items 10 through 18 pertain to hyperactivity-impulsivity), and an additional 8 items (19-26) related to symptoms of oppositional defiant disorder, as delineated in the *DSM-IV-TR* (*Diagnostic and Statistical Manual of Mental Disorders* [Fourth Edition, Text Revision]). Each item is rated on a 3-point Likert scale.

The Conners Rating Scale was initially developed in 1969 and has undergone several revisions since. It has gained widespread acceptance in both clinical and research settings as a tool for screening, diagnosing, and evaluating treatment efficacy for ADHD and associated symptoms. CPRS-48 is tailored to assess ADHD and related behavioral issues in children and adolescents, ranging in age from 3 to 17 years [18]. The CPRS-48, a derivative of earlier versions, consists of 48 questions answered on a 4-point scale: 0 for normal, 1 for mild, 2 for moderate, and 3 for severe, with higher scores indicating greater severity of behavioral issues. The revised scale uses 6 subscales—conduct problems, learning problems, psychosomatic problems, impulsive-hyperactive, anxiety, and attention index—to evaluate an array of behavioral outcomes.

VRAT used a head-mounted display (HMD) as part of a VR system to assess attention processes. Participants were seated at a desk and a physician fitted the HMD to each child’s head before activating the system, which presented a virtual classroom environment. Participants wore HMDs that immersed them in a simulation of a typical rectangular classroom with 3 rows of desks, a teacher’s desk and blackboard at the front, a female virtual teacher seated in between the desk and the blackboard, a sizable window on the left side showing a playground with buildings, vehicles, and people, and doorways on the opposite wall through which various activities took place. Participants received directions from the virtual instructor. They were instructed to keep an eye on a series of letters displayed on the blackboard and to quickly hit a response button only when the letter “X” was followed by the letter “A.” Then, before beginning the real testing, the technician led the participants in a brief exploration session during which they were encouraged to look about the virtual room and identify various objects until they familiarized themselves with the VRAT environment.

**Figure 1.**
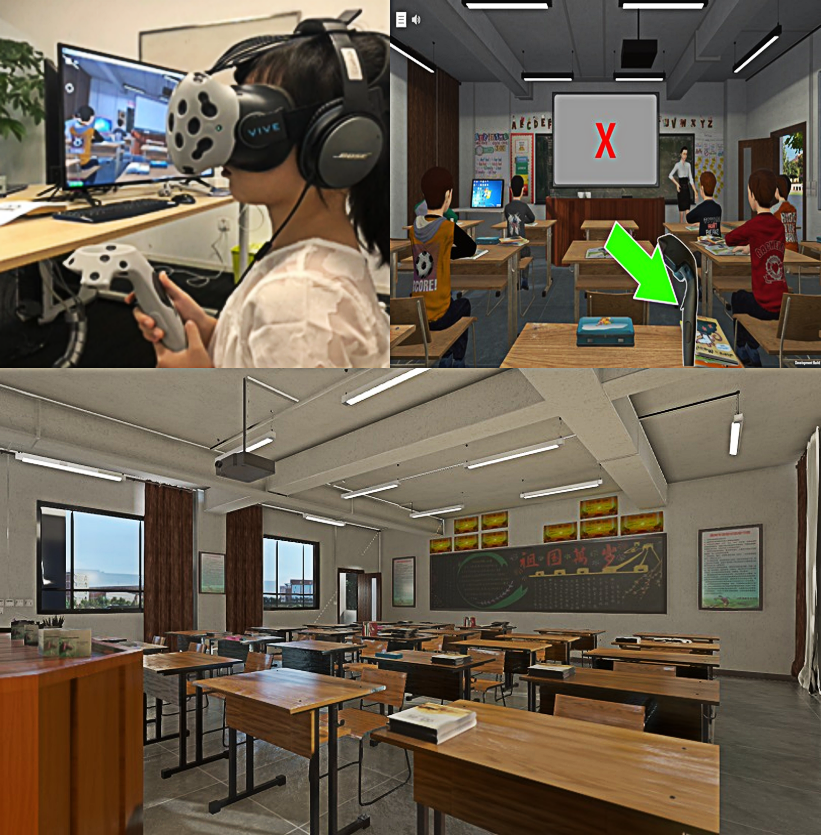
Virtual Reality Attention Tracker testing environment for attention-deficit/hyperactivity disorder assessment in school-aged children.

The stimuli presented included the letters A, B, C, D, E, F, G, H, J, L, and X. These letters were displayed at a rate of one every 1350 ms, remaining visible for 150 ms. The entire testing session lasted 13 minutes and contained 520 stimuli. The target letter “X” (which comes after an “A” before it) occurs 52 times in total (10% of stimuli). The task stimuli are balanced across 4 blocks (13 targets per block). During this task, the virtual classroom was filled with a multitude of distractors, including auditory (eg, pencils dropping and footsteps), visual (eg, a paper airplane soaring across the room), and mixed (both auditory and visual, such as a bus passing by the outside window). Each distractor was visible for 5 seconds and occurred at randomly assigned intervals of 10, 15, or 25 seconds. In total, there were 36 distractors, with 12 different types presented 3 times each. As the virtual classroom task unfolded, data were recorded for each block, capturing the number of correct hits (instances where a response coincided with the target “AX” sequence) and commission errors (instances where a response was made to a nontarget). By incorporating a dynamic and immersive virtual environment, the VRAT aims to assess attention and impulsivity under conditions that closely mimic real-life classroom settings. This is anticipated to provide more ecologically valid data regarding the attentional processes of children, particularly those with ADHD (Figure 1).

Illustration of the virtual classroom environment used in the VRAT for assessing attention and hyperactivity in a cross-sectional study of 425 school-aged children (aged 6‐8 years) at Shenzhen Hongling Primary School, Shenzhen, Guangdong Province, China, conducted from November 11 to December 10, 2022. The setup includes an HMD presenting a simulated classroom with a teacher, desks, a blackboard, and distractors (eg, auditory and visual stimuli) to evaluate ADHD-related behaviors.

### Key Performance Variables of VRAT

**Figure 2.**
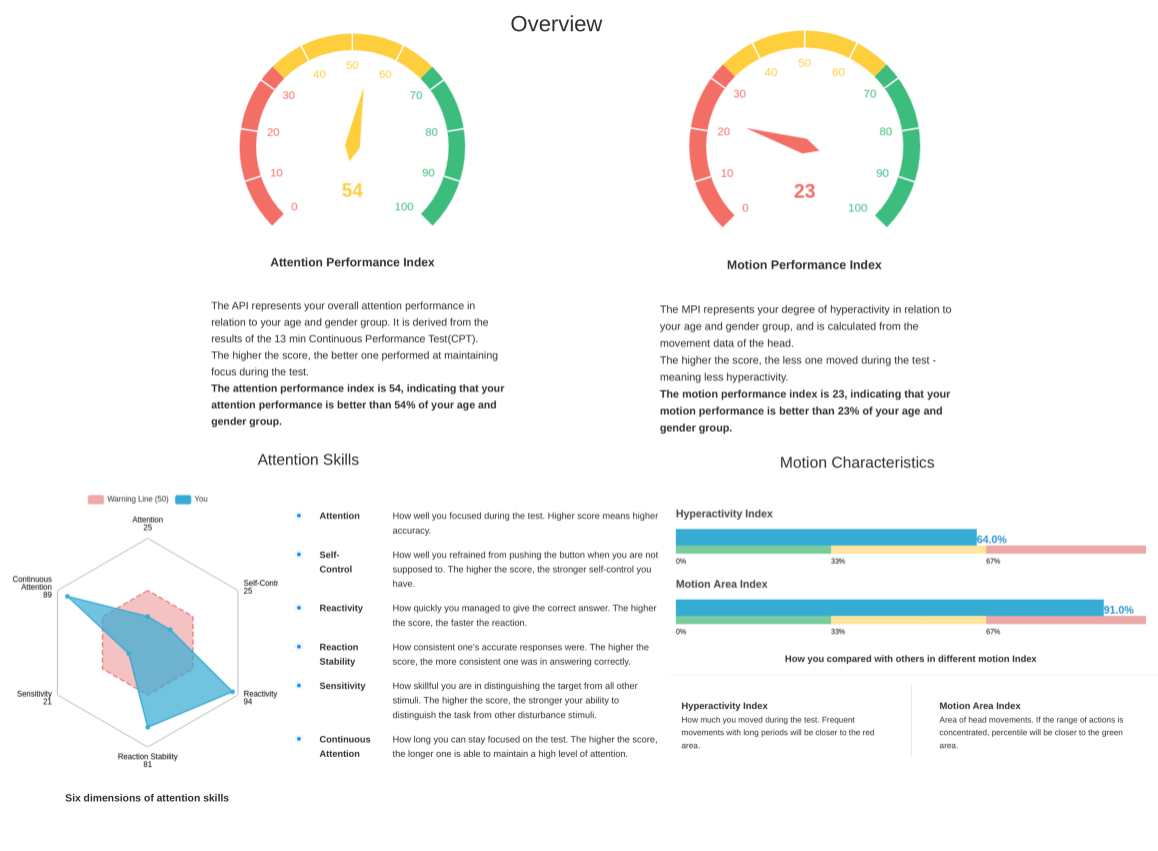
Illustrative example of key performance variables of the Virtual Reality Attention Task in the report.

The key performance variables assessed in VRAT comprised 2 primary indices: the attention performance index and the motion performance index. The attention skills encompassed 6 core attentional domains: attention, self-control, reactivity, reaction stability, sensitivity, and continuous attention. The motion characteristics included the hyperactivity index and motion area index. The following (Figure 2 and Textbox 2) is a description of the different measures obtained with VRAT.

Textbox 2. **The descriptions of the different measures in the Virtual Reality Attention Task (VRAT).**

- Attention performance index: This index represents the overall attention performance and is derived from the composite results of the 13-minute continuous performance tests. A higher score indicates better performance in maintaining focus during the test.
- Motion performance index: This index quantifies the degree of hyperactivity, based on movement data of the head and hand. A higher score signifies less movement during the test, indicating reduced hyperactivity.
- Attention (omission errors): These errors occur when the participant fails to respond to the target stimulus as required. Omission errors are indicative of challenges in selective and focused attention.
- Self-control (commission errors): Commission errors occur when the participant presses the button in the absence of a target stimulus. These errors reflect deficiencies in motor control or response inhibition.
- Reactivity (latency): This measures the average response time to the target.
- Reaction stability (COV): Coefficient of variation of children’s reaction time, COV=variability/mean (latency)*100
- Sensitivity (D-prime): D-prime measures the participant’s ability to discriminate between signal and noise. It is calculated as a Z-score, providing a standardized metric for evaluating changes in subsequent test sessions.
- Continuous attention (continuity): This measures the stability of response accuracy across 4 time intervals.
- Hyperactivity index (immobility duration inversion): This is a quantitative measure where a higher score indicates a greater level of hyperactive-impulsive behavior. It is inversely related to the immobility duration (the total time a child remains still). This means that a shorter immobility duration will result in a higher hyperactivity index score, and vice versa.
- Motion area index (area): This represents the area covered by the path of the sensor.

### Data Analysis

Data following a normal distribution were described using means and SDs. Participants scoring more than 3 SD from the mean on any variable were excluded from that variable’s analysis. Normality was assessed using the Shapiro-Wilk test. In cases of high skewness, data transformation was applied (reciprocal transformation for commission error rate and log-transformation for head position shift and rotation). For the receiver operating characteristic (ROC) analysis, the original “Immobility Duration” and “Area” metrics were also transformed using their reciprocal values (“1/Immobility” Duration and “1/Area”). This was done to ensure that, consistent with all other VRAT indices, thereby aligning the directional interpretation of all metrics in the comparative analysis. Mann-Whitney *U* tests evaluated gender differences. Pearson correlation coefficients assessed relationships between VRAT and SNAP-IV and CPRS-48 scores. For ROC analysis, “at-risk” versus “healthy” labels were defined using SNAP-IV cutoffs (>1.5 for inattention or hyperactivity-impulsivity), chosen for their alignment with *DSM-5* (*Diagnostic and Statistical Manual of Mental Disorders* [Fifth Edition]) criteria and established psychometric properties [3]. This approach, while practical for a nonclinical sample, lacks an independent clinical diagnosis, limiting diagnostic specificity. Area under the curve (AUC) was calculated to evaluate VRAT’s discriminative capacity against SNAP-IV labels. Analyses were conducted using SPSS (version 26; IBM Corp).

### Ethical Considerations

This study received approval from the Ethics Committee of The Eighth Affiliated Hospital, Sun Yat-Sen University (2021-044-01). Participation in the study was limited to children whose parents or guardians provided informed consent. The parents were asked to complete a demographic questionnaire, the SNAP-IV questionnaire, and the CPRS-48 scale. Each child completed the VRAT under the supervision of a research assistant who was specifically trained to administer the tasks in a standardized manner. This ensured consistency in the testing environment and procedure for all participants.

No compensation was offered to participants for their involvement in this study. The study was conducted with the understanding that the contribution of the participants was purely for the advancement of scientific knowledge and the public good, without any financial incentives. In addition to the absence of financial compensation, the study ensured the anonymity of all participants by removing any personally identifiable information from the data collected.

## Results

### Overview

**Figure 3.**
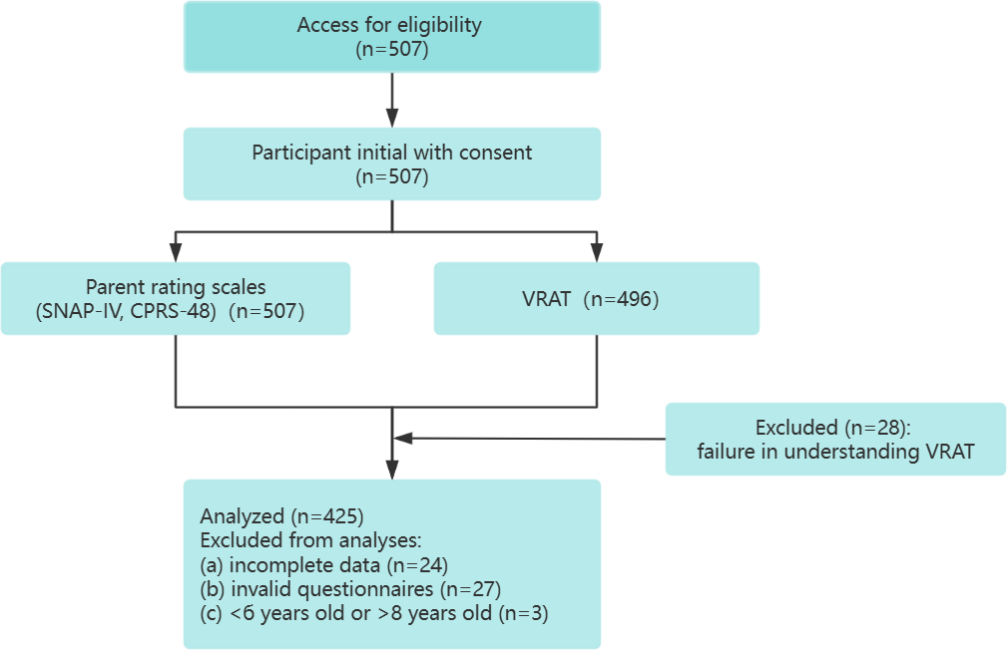
Flowchart of participant recruitment and assessment process for attention-deficit/hyperactivity disorder evaluation. SNAP-IV: the Swanson Nolan and Pelham, Version IV Scale-Parent Scales; VRAT: Virtual Reality Attention Tracker; CPRS-48: the Chinese version of the Conners’ Parent Rating Scale–Revised.

A total of 507 participants were selected from the elementary school and then 82 participants were excluded due to invalid questionnaires, incomplete data, or difficulties in comprehending the VRAT or completing practice trials. The final sample comprised 425 participants, with an equal distribution of 236 boys (55.53%) and 189 girls (44.47%), aged between 6 and 8 years (mean 6.724, SD 0.66; Table 1 and Figure 3).

**Table 1.** Demographic characteristics of participants in a cross-sectional attention-deficit/hyperactivity disorder study.

Characteristics	Value
Age (years), mean (SD)	6.724 (0.66)
Gender, n (%)	
Male	236 (55.53)
Female	189 (44.47)
Parental education level, n (%)	
Primary	45 (10.59)
Secondary	230 (54.12)
Tertiary	150 (35.29)

Demographic characteristics of 425 school-aged children (aged 6‐8 years) recruited from Shenzhen Hongling Primary School, Shenzhen, China, for a cross-sectional study of ADHD using the VRAT, SNAP-IV, and CPRS-48, conducted from November 11 to December 10, 2022. Of the 507 participants, 425 completed both parent rating scales (SNAP-IV and CPRS-48) and the VRAT after exclusions due to invalid questionnaires (n=82), incomplete data, or comprehension difficulties.

### Association Between the VRAT and the SNAP-IV

There were significant correlations between VRAT and SNAP-IV variables. Participants with higher inattention scores on the SNAP-IV tended to have scores lower on the attention performance index, motion performance index, omission errors, commission errors, coefficient of variation (COV), D-prime, and continuity (all *P*<.001). Furthermore, hyperactivity-impulsivity scores on SNAP-IV were negatively correlated with the attention performance index, motion performance index, omission errors, commission errors, D-prime and continuity (all *P*<.001), latency and COV (both *P*<.01). Significant positive correlations were observed between the immobility duration and area measured in the VRAT and both inattention scores and hyperactivity-impulsivity scores on the SNAP-IV scale (all *P*<.001; Table 2).

**Table 2.** Correlations between Virtual Reality Attention Tracker measures and SNAP-IV subscales in school-aged children.

VRAT^a^ and SNAP-IV^b^	Inattention		Hyperactivity-impulsivity	
	*r*	*P* value	*r*	*P* value
Attention performance index	–0.28	<.001	–0.27	<.001
Motion performance index	–0.30	<.001	–0.38	<.001
Omission errors	–0.23	<.001	–0.22	<.001
Commission errors	–0.21	<.001	–0.20	<.001
Latency	–0.07	.136	–0.13	.008
COV^c^	–0.18	<.001	–0.16	.001
D-prime	–0.28	<.001	–0.25	<.001
Continuity	–0.22	<.001	–0.22	<.001
Immobility duration	0.26	<.001	0.35	<.001
Area	0.27	<.001	0.31	<.001

a VRAT: Virtual Reality Attention Tracker.

b SNAP-IV: the Swanson Nolan and Pelham, Version IV Scale-Parent Scales.

c COV: coefficient of variation of children’s reaction time, COV=variability/mean (latency)*100, COV=variability/mean (latency)*100. variability/mean (latency)*100.

### Association Between the VRAT and the CPRS-48

As summarized in Table 3, there were no significant correlations between the latency scores in the VRAT and all CPRS-48 scores (all *P*>.05). And there were also no significant correlations between the anxiety scores in the CPRS-48 and all VRAT scores (all *P*>.05). With regard to the COV scores in the VRAT, conduct problems scores (*r*=−0.086; *P*=.08), psychosomatic problems scores (*r*=−0.081; *P*=.09) showed no statistically significant correlations. And there was no correlation observed between commission error scores in VRAT and psychosomatic scores in CPRS-48 (*r*=−0.078; *P*=.11). In addition to the above variables, the other variables of VRAT and CPRS showed significant correlations (all *P*<.05).

**Table 3.** Correlations between Virtual Reality Attention Tracker measures and the Chinese version of the Conners’ Parent Rating Scale–Revised (CPRS-48) subscales in school-aged children.

VRAT^a^CPRS-48^b^	Conduct problems		Learning problems		Psychosomatic problems		Impulsivity-hyperactivity		Anxiety		Attention index	
	*r*	*P* value	*r*	*P* value	*r*	*P* value	*r*	*P* value	*r*	*P* value	*r*	*P* value
Attention performance index	–0.18	<.001	–0.28	<.001	–0.13	.007	–0.16	.001	–0.07	.14	–0.22	<.001
Motion performance index	–0.22	<.001	–0.25	<.001	–0.10	.03	–0.22	<.001	–0.01	.90	–0.26	<.001
Omission errors	–0.14	.003	–0.22	<.001	–0.13	.008	–0.10	.04	–0.05	.32	–0.16	.001
Commission errors	–0.13	.006	–0.20	<.001	–0.08	.110	–0.14	.003	–0.01	.86	–0.16	.001
Latency	–0.07	.18	–0.09	.08	–0.05	.35	–0.02	.64	–0.07	.16	–0.09	.080
COV^c^	–0.09	.08	–0.18	<.001	–0.08	.094	–0.11	.030	–0.05	.34	–0.14	.003
D-prime	–0.17	<.001	–0.27	<.001	–0.13	.007	–0.15	.002	–0.05	.35	–0.20	<.001
Continuity	–0.17	<.001	–0.24	<.001	–0.18	<.001	–0.13	.009	–0.08	.09	–0.18	<.001
Immobility duration	0.19	.001	0.21	<.001	0.11	.025	0.18	<.001	–0.02	.72	0.22	<.001
Area	0.19	<.001	0.21	<.001	0.18	.027	0.19	<.001	–0.02	.64	0.21	<.001

a VRAT: Virtual Reality Attention Tracker.

b CPRS-48: the Chinese version of the Conners’ Parent Rating Scale–Revised.

c COV: Coefficient of variation of children’s reaction time, COV=variability/mean (latency)*100.

### Differences by Gender

Participants were categorized into 2 groups based on gender (236 boys and 189 girls). Mann-Whitney *U* tests were conducted to examine the mean differences in VRAT, SNAP-IV, and CPRS-48 scores, to determine whether performance varied across the tests. In terms of gender differences for the SNAP-IV, statistically significant differences were observed in both inattention and hyperactivity-impulsivity (all *P*<.01). Additionally, statistically significant differences were found for conduct problems scores, learning problems scores, impulsivity-hyperactivity scores, and attention index scores in boys who showed higher scores than girls in CPRS-48 (all *P*<.001). In the VRAT, boys performed significantly worse than girls in latency (all *P*<.01), as illustrated in Table 4.

**Table 4.** Gender differences in Virtual Reality Attention Tracker, SNAP-IV, and CPRS-48 scores in school-aged children.

	Boys (n=236)	Girls (n=189)	U value	*P* value
VRAT^a^, mean (SD)				
Attention performance index	63.62 (16.21)	63.94 (15.64)	–0.042	.97
Motion performance index	61.53 (23.46)	66.31 (19.44)	1.869	.06
Omission errors	67.89 (25.47)	68.54 (25.70)	0.203	.84
Commission errors	73.27 (21.10)	71.16 (22.86)	–0.831	.41
Latency	41.61 (26.16)	49.47 (24.57)	3.244	<.001
COV^b^	65.28 (21.70)	60.16 (26.96)	–1.575	.12
D-prime	72.75 (22.12)	72.87 (23.44)	0.531	.60
Continuity	69.76 (22.43)	67.79 (24.07)	–0.827	.41
Immobility duration	0.38 (0.26)	0.33 (0.21)	–1.793	.07
Area	0.38 (0.28)	0.33 (0.24)	–1.112	.27
SNAP-IV^c^				
Inattention	0.80 (0.51)	0.65 (0.40)	–2.982	.003
Hyperactivity-impulsivity	0.67 (0.52)	0.39 (0.32)	–5.77	<.001
CPRS-48^d^				
Conduct problems	0.43 (0.38)	0.30 (0.26)	–3.341	<.001
Learning problems	0.61 (0.45)	0.48 (0.41)	–2.742	.006
Psychosomatic problems	0.18 (0.28)	0.17 (0.23)	0.524	.60
Impulsivity-hyperactivity	0.54 (0.46)	0.36 (0.33)	–3.641	<.001
Anxiety	0.32 (0.31)	0.33 (0.27)	0.974	.33
Attention index	0.51 (0.41)	0.36 (0.29)	–3.685	<.001

a VRAT: Virtual Reality Attention Tracker.

b COV: coefficient of variation of children’s reaction time, COV=variability/mean (latency)*100. Coefficient of variation of children’s reaction time, COV=variability/mean (latency)*100.

c SNAP-IV: the Swanson Nolan and Pelham, Version IV Scale-Parent Scales.

d CPRS-48: the Chinese version of the Conners’ Parent Rating Scale–Revised.

### Sensitivity and Specificity

In order to further examine the similarities and differences between the different assessment tools, a ROC analysis was performed. The ROC curve plots the true positive rates (sensitivity) against false positive rates (1 – specificity). The current dataset contained no individuals diagnosed with ADHD; therefore, classification labeling was defined as “at-risk” versus “healthy” according to their assessment scores on SNAP-IV. SNAP-IV scores were chosen for labeling as they follow the *DSM-5* diagnostic criteria and provided cutoff scoring references. Children were considered to be at risk for ADHD if they obtained a SNAP-IV hyperactivity-impulsivity score >1.5 or an inattention score >1.5, or both (the suggested target score for nonclinically significant symptoms is an averaged inattention or hyperactivity-impulsivity score of 0‐1; the suggested target scores for at-risk, moderate, and severe symptoms are 1.1‐1.5, 1.6‐2, and 2‐3, respectively).

**Figure 4.**
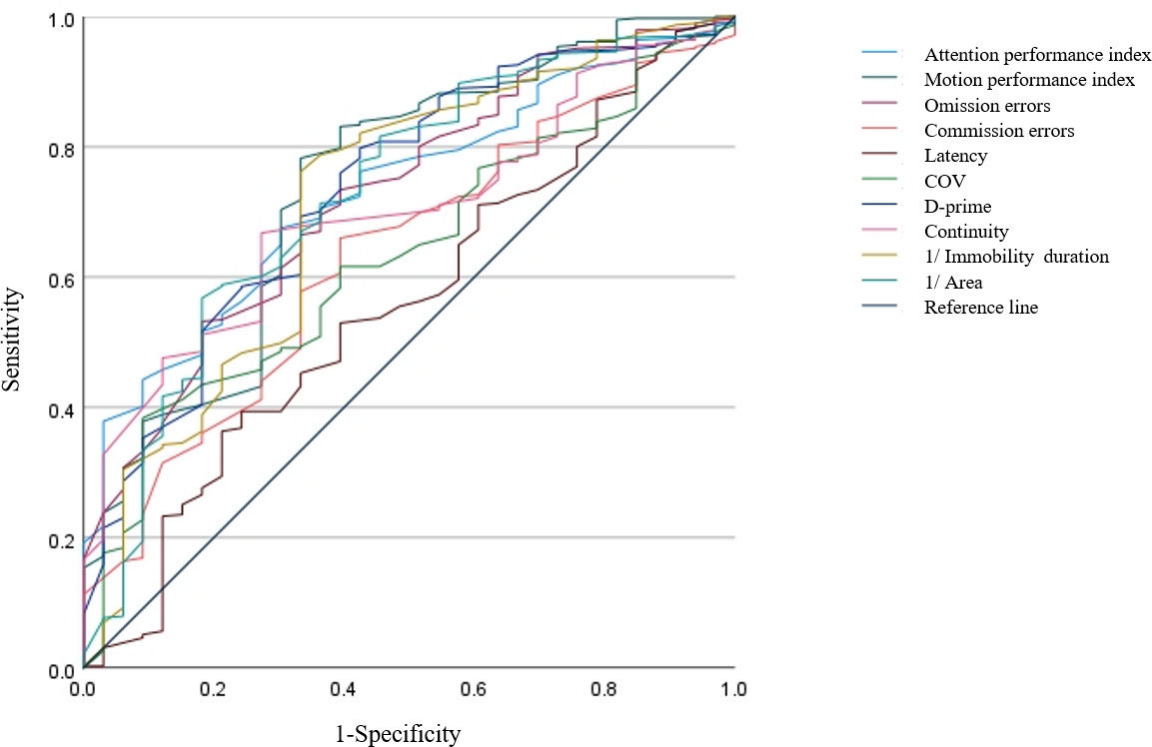
Receiver operating characteristic curves for Virtual Reality Attention Tracker in differentiating at-risk attention-deficit/hyperactivity disorder symptoms. COV: coefficient of variation of children’s reaction time. COV = variability/mean (latency) * 100.

Using the binary outcome labels derived from SNAP-IV scores (at-risk vs healthy), the similarity between VRAT and SNAP-IV was determined by calculating the ROC curve of VRAT variables as continuous predictors of the binary outcome. The AUC is considered a summary measure of the ROC curve and was used to evaluate the classification similarity between VRAT and SNAP-IV. AUC was calculated for all VRAT variables; of these variables, the attention performance index, motion performance index, omission errors, D-prime, and “1/Area” showed the highest similarity to the SNAP-IV labeling (AUC=0.73; AUC=0.74; AUC=0.73; AUC=0.73, respectively), corroborating the results from the correlation analysis where these variables showed the highest *r* coefficients. The metrics “Immobility Duration” and “Area” are presented as their reciprocal values (“1/Immobility Duration” and “1/Area”) to ensure consistent directional interpretation with other indices (Table 5 and Figure 4).

**Table 5.** Discriminative validity of Virtual Reality Attention Tracker subscales in identifying at-risk ADHD^a^ symptoms in school-aged children.

Index	AUC^b^	Cutoff	Sensitivity (%)	Specificity (%)
Attention performance index	0.73	58.50	67.52	69.70
Motion performance index	0.74	48.50	78.26	66.67
Omission errors	0.73	74.00	53.20	81.82
Commission errors	0.63	70.00	65.98	60.61
Latency	0.56	51.00	39.39	75.76
COV^c^	0.63	75.50	38.36	90.91
D-prime	0.73	56.50	79.80	57.58
Continuity	0.70	62.50	66.75	72.73
1/Immobility duration	0.71	1.87	76.21	66.67
1/Area	0.73	2.90	56.78	81.82

a ADHD: attention-deficit/hyperactivity disorder.

b AUC: area under the curve.

c COV: coefficient of variation of children’s reaction time, COV=variability/mean (latency)*100.

## Discussion

### Principal Findings

This study aimed to evaluate the convergent validity of the VRAT by examining its associations with parent-rated ADHD symptoms using the SNAP-IV and CPRS-48 scales, while also assessing gender differences and the VRAT’s discriminative capacity in a nonclinical sample of school-aged children. Key findings include moderate negative correlations between VRAT performance metrics (eg, attention and motion indices) and parent ratings of inattention and hyperactivity-impulsivity, minimal gender differences in VRAT scores except for latency, and satisfactory discriminant validity, highlighting VRAT’s potential as an ecologically valid adjunct to traditional ADHD assessments.

The observed correlations between VRAT metrics and SNAP-IV and CPRS-48 subscales indicate that VRAT effectively captures objective manifestations of ADHD-related deficits in attention and motor control, which suggest that children with more head movement in the virtual classroom are likely to show signs of hyperactivity and have trouble focusing in daily life [18,19]. Previous studies have indicated that conventional neuropsychological assessments often exhibit limited associations with ADHD rating scale outcomes [20,21]. The modest relationship between CPT performance and behavioral ratings may be attributed to the distinct aspects of behavior assessed by computerized tasks and self-report scales, making it challenging to fully capture ADHD symptoms through a single measure [22,23]. Traditional CPTs also struggle to measure distractibility in real-world settings, a gap addressed by VRAT, which enhances ecological validity through immersive classroom simulations [24,25]. VRAT improves engagement, reduces bias, and enables more reliable cognitive assessments by providing a standardized environment [26]. While ADHD is linked to social and academic challenges, our study found no correlation between VRAT scores and CPRS-48 anxiety symptoms, consistent with previous research suggesting CPTs have limited utility in distinguishing ADHD from mood and anxiety disorders [27,28].

Gender differences were pronounced in parent ratings, with boys scoring higher on SNAP-IV inattention and hyperactivity-impulsivity (*P*<.01) and CPRS-48 subscales (eg, conduct problems, impulsivity-hyperactivity; *P*<.001), consistent with literature on males’ tendency toward externalizing behaviors [29,30]. In contrast, VRAT metrics showed minimal gender differences, except for latency, where girls exhibited longer response times (*P*<.01). This divergence may reflect VRAT’s focus on objective neurocognitive processes, which are less influenced by gender-specific behavioral phenotypes or parental reporting biases, where boys’ hyperactive symptoms are overrated [31,32].

The minimal gender differences observed in our primary VRAT analysis suggest that the tool may capture core attentional processes that are less gender-divergent in this specific context, which is consistent with prior research showing reduced gender divergence in objective ADHD measures [29]. However, this finding must be interpreted in light of compelling evidence that girls often present with subtler, nonhyperactive profiles that are easily overlooked and contribute to underidentification [33]. Our results, which show a general absence of large motoric differences, could be consistent with this narrative of a less conspicuous manifestation. It is possible that the immersive, ecologically valid nature of the VRAT task mitigated the more overt motoric differences often seen in traditional settings, thereby capturing a profile where gender disparities are less pronounced. Within this context, the longer latency in girls may indicate compensatory cognitive strategies, differential maturation of processing speed, or measurement artifacts unrelated to ADHD [34,35]. This underscores the critical need for future research with larger, longitudinally designed samples to disentangle the complex interplay of age, assessment modality, and cultural context in shaping the expression of ADHD-related behaviors across genders.

ROC analysis revealed satisfactory discriminant validity for VRAT metrics (AUC=0.56‐0.74), particularly for attention performance index, motion performance index, omission errors, D-prime, and “1/Area” (AUC≥0.73). Omission errors in VRAT manifest when participants neglect to respond to the designated target stimulus, suggesting difficulties in selective and focused attention and serving as a dependable indicator for predicting ADHD [26,36]. Conversely, commission errors occur when participants erroneously press a button in the absence of a target stimulus, reflecting impairments in motor control or response inhibition and demonstrating strong correlations with ADHD [37]. D-prime, on the other hand, assesses the participant’s capacity to differentiate between signal and noise, enabling the classification of children with and without ADHD [38]. The analysis of head movement variables in VRAT offers insight into behaviors associated with hyperactivity and distraction, showing promise in predicting ADHD.

### Comparison to Prior Work

Our findings align with prior studies highlighting the ecological validity of VR-based assessments for ADHD. Adams et al [39] reported a superior classification rate for a virtual classroom task (87.5%) compared to traditional CPTs (68.8%), consistent with our ROC results (AUC=0.73‐0.74 for key VRAT metrics). The VRAT’s immersive classroom environment, incorporating auditory and visual distractors, addresses limitations of traditional CPTs, which often fail to capture real-world distractibility [24,25]. Our study provides a direct evaluation of the correlations between the VRAT and parent-rated scales, reinforcing its ability to mirror behavioral observations in a clinical setting [16]. This approach allows for a clear interpretation of the VRAT’s ecological validity. Furthermore, our findings highlight the specificity of the VRAT for ADHD-related symptoms. This is evidenced by the lack of significant correlation with CPRS-48 anxiety scores (*P*>.05 for all comparisons), a finding that diverges from studies such as Solanto et al [28], which reported limited utility for traditional CPTs in distinguishing ADHD from mood disorders [27].

### Limitations

It is imperative to highlight certain limitations of the study. First, the study used a nonclinical sample of 425 school-aged children without confirmed ADHD diagnoses, limiting the assessment of VRAT’s diagnostic accuracy in clinical populations. This may have reduced the observed effect sizes, as symptom severity is typically higher in clinical cohorts [40]. To mitigate this, we applied strict inclusion and exclusion criteria and used validated SNAP-IV cutoffs to define “at-risk” groups. Future studies should validate VRAT in clinically diagnosed ADHD populations to confirm its sensitivity and specificity. Second, reliance on parent-reported SNAP-IV and CPRS-48 scales introduces potential rater bias, as parental perceptions may be influenced by gender stereotypes or situational factors. We mitigated this by excluding outliers (eg, questionnaires taking >30 minutes) and using standardized scoring protocols. However, this may have led to underreporting or overreporting of symptoms, particularly for girls, who exhibited lower scores on rating scales. Incorporating teacher ratings or clinical interviews in future studies would enhance multi-informant validity [41,42]. Third, the external validity of our findings is constrained by the convenience sampling method. All participants were recruited from a single public primary school in an urban area of a major first-tier city in China. While this homogeneous sample aids internal validity, it limits the generalizability of the results to populations from rural areas, other types of schools, or different cultural contexts. Future multisite studies encompassing diverse geographic and demographic settings are warranted to confirm the broad applicability of our findings.

### Future Directions

Future research should validate VRAT in clinically diagnosed ADHD cohorts to assess its diagnostic accuracy and explore its applicability across ADHD subtypes (eg, inattentive, hyperactive-impulsive, and combined). Longitudinal studies are needed to evaluate whether VRAT performance predicts real-world functional outcomes, such as academic performance or treatment response. Incorporating multi-informant assessments (eg, teacher ratings and clinical interviews) would strengthen VRAT’s ecological validity. In addition, expanding VRAT to include modules for other executive functions (eg, working memory and cognitive flexibility) could enhance its utility as part of a comprehensive ADHD assessment battery. Comparative studies with other VR-based tools, such as Aula Nesplora, would clarify VRAT’s unique contributions in terms of effect sizes and technical requirements.

### Conclusions

This study demonstrates that the VRAT is a promising tool for objectively assessing ADHD-related attention and hyperactivity behaviors in school-aged children, with significant correlations to parent-rated SNAP-IV and CPRS-48 scores and satisfactory discriminant validity (AUC=0.56‐0.74). The VRAT’s immersive environment enhances ecological validity compared to traditional CPTs, capturing real-world distractibility. However, its reliance on a nonclinical sample and parent reports highlights the need for complementary assessment methods to ensure comprehensive ADHD evaluation.

## Abbreviations

ADHD: attention-deficit/hyperactivity disorder
AUC: area under the curve
COV: coefficient of variation
CPRS-48: the Chinese version of the Conners' Parent Rating Scale–Revised
CPT: continuous performance test
*DSM-5*: *Diagnostic and Statistical Manual of Mental Disorders* (Fifth Edition)
*DSM-IV-TR*: *Diagnostic and Statistical Manual of Mental Disorders* (Fourth Edition, Text Revision)
HMD: head-mounted display
ROC: receiver operating characteristic
SNAP-IV: the Swanson Nolan and Pelham, Version IV Scale-Parent Scales
VR: virtual reality
VRAT: Virtual Reality Attention Tracker
